# No additional copies of HERV-Fc1 in the germ line of multiple sclerosis patients

**DOI:** 10.1186/1743-422X-9-188

**Published:** 2012-09-08

**Authors:** Kari K Nissen, Magdalena J Laska, Bettina Hansen, Finn S Pedersen, Bjørn A Nexø

**Affiliations:** 1 Department of Biomedicine, Aarhus University, Aarhus, Denmark; 2 Department of Molecular Biology and Genetics, Aarhus University, Aarhus, Denmark

**Keywords:** HERV-Fc1, Multiple sclerosis, Provirus copy-number

## Abstract

**Background:**

Human endogenous retroviruses (HERVs) are suspected to play a role in the development of multiple sclerosis (MS). This suspicion has in part been based on increased expression of viral RNA or proteins or antibodies targeting retroviral products in MS patients. Recently, our group provided genetic evidence for association between the endogenous retrovirus HERV-Fc1 and MS, suggesting that HERV-Fc1 plays a role in this multifactorial disease. We have found increased expression of HERV-Fc1 in MS patients suffering from recent attack, but the underlying mechanism for association is still unknown.

**Findings:**

Evidence from animal models indicates that ERV implication in the pathogenesis of diseases can be a result of extra copies of the virus in the germ line. Therefore, we investigated the genome of 81 individuals, 74 patients with MS and 7 healthy controls, by means of Southern blotting, for presence of extra HERV-Fc1 copies. The known insertion at the Xq21.33 position was readily detectable, but no additional insertions in other genomic contexts could be identified in any studied individuals. This substantiates our previous copy-number PCR findings of a 2:1 ratio of HERV-Fc1 DNA between women and men, as expected from the X-chromosome location; there was no difference between patient and control individuals.

**Conclusions:**

No additional germ line copies of HERV-Fc1 could be identified, precluding such copies to underlie the association between this provirus and multiples sclerosis.

## Findings

### Background

Retrovirus-like elements including human endogenous retroviruses (HERVs) occupy around 8 percent of the human genome [1]. Most of these ancient viral elements are disrupted by accumulated mutations and are therefore generally considered harmless. However, about 50 HERVs retain coding potential [2], and a realization of their potential in pathogenesis is growing. Many studies have thus presented different HERVs, especially of HERV-W and HERV-H descent, to be functionally associated with multiple sclerosis (MS), a multifactorial disease with both environmental and genetic factors involved, most of which are still obscure. These associations have been described as increased expression of viral RNA or proteins from HERVs or increased load of host antibodies targeting these have been demonstrated in MS patients [3-6]. For ubiquitous genomic elements like HERVs, the cause-or-consequence relation of such functional correlations can be hard to establish [7]. By investigating HERVs from a genetic viewpoint, exploiting genotype variations, it is possible to identify associations, which more certainly precede the disease development. It is unlikely that a disease can alter the organism’s sequence of genomic, polyclonal DNA.

Recently, we presented genetic association with MS for the HERV-Fc1 locus located on Xq21.33 [8]. One marker neighboring HERV-Fc1 (rs391745) had a p-value of 1.3*10^-6^ for disease association and other markers in and around the provirus were also associated with MS. A pathogenic background for this association has not been established, although we demonstrated an average 4 times increased expression of extracellular HERV-Fc1 *gag* RNA in plasma from MS patients with recent attacks compared to patients in remission or control persons [9]. Additional studies have shown the HERV-Fc1–MS association to hold only for the common “bout onset” subtypes of sclerosis, comprising the remitting/relapsing and secondary progressive forms. No association was found for the rarer primary progressive subtype [10]. This is particularly interesting in the light of bout onset subtypes having an overweight of female patients, whereas the primary progressive subtype has equal gender rates. HERV-Fc1 is located on the X chromosome and X-dominant diseases are known to be more common among females.

In the present study we were inspired by findings in mice, where disease association with endogenous retrovirus has been shown to correlate with increased copy-numbers of the specific virus in the germ line. E.g. the inherited AKR murine leukemia virus was present in more copies in high-virus-yielding mouse strains, with high leukemia incidence, compared to low- or non-virus-yielding mouse strains with low leukemia risk [11]. Therefore, we speculated whether a similar mechanism could account for the HERV-Fc1 involvement in MS.

To date, the HERV-K family is the only family found to have integrations specific to human [12]. Some HERV-K viruses actually occur polymorphically in the human genome; the majority in a study population had the preintegration sites, whereas 30% and 15% of the individuals carried the integrated HERV-K113 and HERV-K115, respectively [13]. Additional reports on retroviral elements occurring polymorphic in the human genome continue to show [14], signifying relatively recent activity of retroviral integration mechanisms. Other such polymorphisms might exist, yet undiscovered due to rare occurrence.

HERV-Fc1, which is part of the enlarged HERV-H/F family, is also an evolutionarily recent genomic acquisition, present only in the genomes of human, chimpanzee, and gorilla [15]. HERV-Fc1 is unusual among human provirus in having only a single known integration in the genome. It is characterized by a relatively preserved sequence with coding-potential for Gag and Env, only three mutations disrupting the *pol* gene, and high LTR identity (94.3%), which argues that the virus could have been recently mobile. Benit et al. have previously searched human genome databases for HERV-Fc elements [15] and found besides the single copy HERV-Fc1 only a related HERV-Fc2 master element of low similarity and some truncated Fc2-like elements. Although belonging to the same family, the HERV-Fc1 and HERV-Fc2 sequences are rather dissimilar, e.g. the *env* sequences show only 65% identity. The HERV-Fc family is thus divided into subgroups, with HERV-Fc1 being the only member of its group.

Putative copies present only in a small part of the population may not be represented in databases. Thus, it remains a possibility that extra copies of HERV-Fc1 segregate in the human population in a minority of individuals, similar to HERV-K.

The objective of this study was to investigate whether HERV-Fc1 association with MS could be due to new, unidentified germ line integrations, somehow contributing to the complex disease development. Differences in the HERV-Fc1 copy numbers could either represent multiple integration events or amplification of the provirus by retrotransposition after the initial integration.

Other groups have dealt with the hypothesis of extra HERV copies, mainly concerning the HERV-W family (hereunder MSRV and ERVWE1) [5,16]. Here, authors reported an increase in HERV-W/MSRV DNA copy number, without evidence of new integration events or viral replication. In contrary, DNA copy numbers of syncytin-1 (env protein of the ERVWE1) were reported to be unchanged. Perron et al. reported the level of copies increased rather noticeably as the disease underwent the development from RRMS to SPMS [5]. Thus, it is unlikely that these findings represent extra germ line copies, rather the possibility of re-integrations in individual cells.

Furthermore, these studies have all made use of quantitative PCR in the copy-number determination. We aimed for a different technique, Southern blotting, which provides qualitative rather than quantitative information on the copy number of a specific genomic sequence.

### Methods, Results, and Discussion

Southern blots on genomic DNA can distinguish even highly similar endogenized proviruses of the same family due to the unique sequences of the flanking DNA.

Peripheral blood mononuclear cells (PBMCs) obtained from MS patients or healthy control persons were kindly provided by T. Christensen, A. Møller-Larsen and T. Petersen. These primary cells were cryopreserved right after sampling, and exposed to no further processing before extraction of genomic DNA using phenol-chloroform. Of a total 81 samples, 74 represented genomic DNA from diagnosed MS patients (45 females, 29 males), and 7 from healthy control individuals (6 females, 1 male). 10 μg DNA was digested with *AflII* enzyme (New England Biolabs, USA) and separated by 0.7% Tris-acetate-EDTA gel electrophoresis. Control digests were performed with *StuI* (NEB). As positive control, 1x, 3x, and 10x copies of *AflII*-digested BAC RP11-1114 M8 was included. The DNA was transferred to a Zeta-probe GT membrane (Biorad, USA) by vacuum suction and prehybridized, hybridized, and washed as previously described [17]. The DNA-probe was amplified from BAC RP11-1114 M8 (Xq21.33 [GenBank:AL354685]) by means of nested PCR amplifying a 403 bp region of the HERV-Fc1 e*nv* gene found to be unique in BLAST-search (Primers; NestedFc1-F: ctccccatctctctggtgc, NestedFc1-R: tgaggaggctggtttctactaag). Prior to hybridization, the probe was α^32^P-dCTP-labeled by means of Prime-it II Random Primer Labeling Kit (Agilent Technologies, USA).

To test the specificity of the reaction, we first hybridized canine DNA (D17 cell line); there was absolutely no reaction to this DNA. HERV-Fc1-like elements have been described in the canine genome [18], but BLAST-searches show minor similarity between the human ERV-Fc1 *env* gene (SB-probe hybridization site) and the canine ERV-Fc1 *env*.

**Figure 1:**
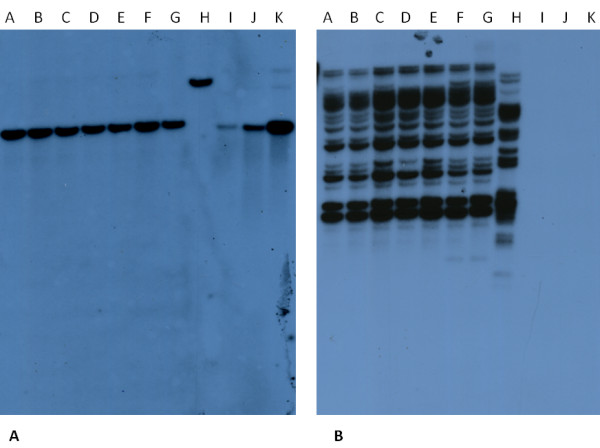
**Representative Southern blot. Seven samples of genomic DNA digested with*AflII*(Lane A-G) or*StuI*(Lane H, same DNA as in G).** Lane I-K = *AflII* digested RP11-1114 M8 BAC 1x, 3x, and 10xcopies respectively. **A** HERV-Fc1 *env* probe, **B** HERV-K family *env* probe. Only one genomic insertion is found for HERV-Fc1, whereas numerous integrated viruses are detected by the HERV-K probe.

A representative Southern blot with human DNAs is presented in Figure 1A. Among the 81 genomic DNAs analyzed, the expected HERV-Fc1 integration at position Xq21.33 was always readily detectable migrating as a 4313 bp band as predicted for *AflII* digestion. Control digests with an alternative enzyme, *StuI*, resulted in predicted migration of 7752 bp, confirming hybridization to the true Fc1 *env* sequence. However, no additional integration sites were identified in any samples.

To verify that we were indeed able to detect several copies of a provirus with many integration sites, we designed a 398 bp probe targeting the *env* gene in viruses of the HERV-K family (BAC RP11-749 M10 (1q23.3, [GenBank:AF012336.2]), primers; HERV-K18-F: gcctcgccatccatccatatt, HERV-K18-R: gcgtctaaccatgtcccagtg). This HERV family has numerous copies in the human genome, and the relative recent integration is reflected in the high sequence similarity of proviruses of this family. The probe had 96-100% homology to at least HERV-K18, -K113, and -K115 but hybridization to a number of additional HERV-Ks was expected and seen (BLAST search identified >10 positions in the human genome with >95% probe-identity), Figure 1B.

The Southern blot method is qualitative rather than quantitative and not suited for actual copy-number estimations based on band intensity. Instead, it is an ideal tool for analyzing the potential integration of viral sequence to unknown chromosomal areas, since new integrations will be apparent from the diverse migration distances. Moreover, although the Southern probe hybridizes to highly homologous sequences, the length of the probe allows some deviation and will therefore recognize related sequences with some variation. This is relevant in the context of endogenized retroviruses, where mutations tend to accumulate in time after integration.

A limitation of this method is the incapacity of detecting un-integrated (circular) virus specimens and duplication of larger chromosomal areas encompassing the external restriction site. In our recent study of HERV-Fc1 expression, the HERV-Fc1 copy-number was analyzed based on quantitative real-time PCR [9]. The DNA analyzed in this context originated from another group of study individuals, including 31 MS patients and 30 healthy control persons. Genomic DNA from all persons derived from PBMCs was amplified and the copy-number estimated from the standard (a linearized plasmid containing the HERV-Fc1 sequence). This study showed an average 9.3x10^3^ HERV-Fc1 *Gag* copies/μl in women and 4.5x10^3^ copies/μl in men (in 40 ng/μl samples), irrespective of patient or control status. The 2:1 ratio between women and men confirms the specificity of the assay from the X-chromosomal location of the HERV-Fc1 locus.

The combined results support the notion that no additional copies of HERV-Fc1 exist. We therefore conclude that extra germ line copies of HERV-Fc1 are not a common phenomenon in MS patients and can thus be ruled out as a potential contributor to the development of multiple sclerosis.

Our main focus has been on MS patients from the perspective that a variation involved in disease development should mainly be represented in this population group. Since the subtype diagnosis was not available for all patients, the samples have not been stratified, but all subtypes were represented in the study. Since HERV-Fc1 is associated genetically to bout onset MS, this subtype is of direct interest. However, it could be argued that the lack of association with primary progressive MS renders it more likely that an endogenous retroviral element similar to HERV-Fc1 but not located on the X chromosome could be involved in this subtype. The control group was matched on geographical and ethnical origin, belonging to an age-interval matching the patient group.

Even though we have not found any new integration in this study, it must be emphasized that our experiments do not exclude non-clonal integrations as a result of replication in somatic cells of the individual person. Also, we cannot exclude the possibility that additional copies of HERV-Fc1 segregate in a very small minority of the population, and could be related to other diseases. If so, it would not be expected to be involved in development of multiple sclerosis. Future studies will investigate alternative mechanism as to how HERV-Fc1 could be a contributor to increased risk of developing multiple sclerosis and potentially other autoimmune diseases.

## Abbreviations

BAC, Bacterial artificial chromosome; HERV, Human endogenous retrovirus; LTR, Long terminal repeat; MS, Multiple sclerosis; RRMS, Relapsing/Remitting multiple sclerosis; SPMS, Secondary progressive multiple sclerosis.

## Competing interests

The authors declare that they have no conflicts of interest.

## Authors’ contribution

KKN carried out the Southern blot analysis, participated in the study design and coordination and drafted the manuscript. MJL contributed to study design and interpretation. BH purified the DNA samples. FSP and BAN conceived of the study, participated in its design and BAN helped to draft the manuscript. All authors read and approved the final manuscript.
